# Therapeutic dendritic cell vaccine preparation using tumor RNA transfection: A promising approach for the treatment of prostate cancer

**DOI:** 10.1186/1479-0556-6-2

**Published:** 2008-01-18

**Authors:** Juliana M Sousa-Canavez, Flavio C Canavez, Kátia RM Leite, Luiz H Camara-Lopes

**Affiliations:** 1 Oncocell Division, Genoa Biotechnology SA, Alameda Ministro Rocha Azevedo, 346, 1^st ^floor, 01410-000, São Paulo, SP, Brazil; 2 Laboratório de Investigação Médica da Disciplina de Urologia da Faculdade de Medicina de USP-LIM 55, São Paulo, SP, Brazil

## Abstract

**Background:**

Early prostate adenocarcinoma can be diagnosed through seric prostate-specific antigen (PSA) screenings. However, a fraction of patients progress to an incurable metastatic disease. Therefore, novel therapies for treating these patients are extremely desirable. Therapeutic vaccines based on Dendritic Cells (DCs) carrying tumor antigens have emerged as a promising strategy to initiate an immune response against tumor cells. These vaccines can be prepared using different methodologies, such as the application of tumor mRNA described in this work.

**Methods:**

Mature and immature DCs were obtained in vitro by adding specific cytokines to monocyte cell cultures. RNA extracted from prostate tumor lineage (LNCAP) was introduced into these cells by electroporation and co-incubation. Transfection success was measured by immunocytochemistry of the PSA expression level in DCs.

**Results:**

Cell surface markers, including CD14, CD80, CD86, CCR7, CD11c, and CD1a, confirmed mature and immature DC phenotypes. Both cell maturation stages were successfully used for RNA introduction as shown by PSA characterization.

**Conclusion:**

Our data support the use of mature and immature DCs for vaccine preparation with either RNA electroporation or RNA co-incubation. The highest efficiency, however, was observed when RNA was delivered by electroporation into mature DCs. Due to in vitro RNA transcription, this method allows small tumors to be used for DC vaccine preparation; it is therefore a promising approach for the treatment of metastatic prostate cancer.

## Background

Prostate adenocarcinoma is the most common malignancy diagnosed in males and the second most common cause of cancer death. In its latest report, the Brazilian National Cancer Institute (INCA) estimated that 47,280 new cases of prostate cancer, or 51 new prostate cancer cases for each 100,000 men, arise in Brazil each year [1]. Because of their population screenings using prostate-specific antigens (PSA), the south and southeastern regions of Brazil are expected to exhibit the highest incidence.

Seric PSA level has aided in the diagnosis of very small prostate tumors. This, in association with radical prostatectomy and radiation therapies, has contributed to increasing the curative indexes [2]. After several years of primary therapy, however, PSA levels can rise even in patients with good outcomes predicted by tumor histological parameters. Roughly one-third of these patients progress to incurable metastatic disease, for which there are few treatment options. The chemotherapy currently used has limited efficacy [3,4]. This lack of treatment has led scientists around the world to search for new therapeutic options [5]; one of these options involves the use of Dendritic Cell (DCs) immunotherapy.

Clinical trails based on DCs have been conducted for the treatment of patients with a variety of tumor types [6-8]. Two of these trials were performed by our group, which developed individualized therapeutic vaccines by fusing allogeneic DCs and neoplastic cells isolated from surgically-removed tumors. We have observed only minor side effects but significant clinical benefits like disease stability in 80% of patients after 2–3 doses of vaccine. The mean survival rate was 13 months for melanoma patients and 6 months for renal cell carcinoma patients [9].

DC immunotherapy based on cell fusion depends on large size tumors (>1 gram) and cannot be applied in most cases of prostate cancer. This barrier does not prevent its use, since different methods of vaccine preparation have been described [6-8]. For prostate cancer, DCs are most commonly primed with entire or partial tissue-specific antigens or tumor-associated antigens. Alternatively, mRNA molecules can be transfected into DCs so that entire proteins will be translated, processed, and presented by MHC on the cell surface. Both methods have already been used in clinical trials and were able to initiate immune response; for a review, see [10].

Here, we compare two methods, electroporation and co-incubation, for transfecting RNA into DCs. The amount of RNA and the DC maturation stage for transfection were also analyzed. The prostate tumor cell line (LNCaP) was chosen for this study because it overexpresses PSA and permits easy DC characterization via immunocytochemistry using anti-PSA antibodies.

## Methods

### Reagents

Culture medium consisted of AIM-V or RPMI-1640 supplemented with fetal bovine serum (FBS), streptomycin, and penicillin (all from GIBCO, Rockville, MD, USA). Recombinant human cytokines like granulocyte macrophage-colony stimulating factor (GM-CSF), interleukin 4 (IL-4), and tumor necrosis factor-α (TNF-α) were purchased from Peprotech Inc. (Rocky Hill, NJ, USA). Ficoll-Hypaque used in cell separation was purchased from Amersham (Piscataway, NJ, USA).

### Dendritic cell culture

As described in Barbuto *et al*. [9], peripheral blood mononuclear cells (PBMC) were collected from informed, consenting, healthy donors through apheresis performed in a Cobe Spectra Blood Cell Separator 7.0 (Cobe, Lakewood, CO, USA) that had been programmed for mononuclear cell collection. Acid citrate dextrose was used as a blood anticoagulant (ratio of 1:8–1:11). Mononuclear cells were separated by density gradient centrifugation (Ficoll-Paque 1,077 g/dl). After three washes with RPMI 1640 medium, mononuclear cells were resuspended in AIM-V at a density of 1.3 × 10^7 ^cells/mL and allowed to adhere to culture flasks for 2 h at 37°C in a humidified incubator. Floating cells were gently removed, and AIM-V containing GM-CSF (50 ng/mL) and IL-4 (50 ng/mL) were added. Flasks were maintained at 37°C in a 5% CO2 humidified incubator for 5 days. For immature DC recovery, cultured cells were harvested on the 5^th ^day. For mature DCs, TNF-α (50 ng/mL) was added to the medium on the 5^th ^day and cultured cells were harvested on the 7^th ^day.

### Cell surface phenotype by flow-cytometric analysis

Determination of phenotype was performed by two-color immunostaining using combinations of FITC- and PE-labeled mAbs directed to human CD14, CD80, CD86, CCR7, CD11c, and CD1a (all purchased from BD-Pharmingen, CA, USA). Corresponding isotype-matched mAbs were used as controls. Cells (1 × 10^6^) were resuspended in PBS containing bovine serum albumin 0.1% and then incubated for 20 min at 4°C with optimal concentrations of monoclonal antibodies. Membrane markers were determined by flow cytometry (FACScalibur, Becton Dickinson Immunocytometry Systems, CA, USA), and data from 10,000 events were analyzed by Cell Quest Pro software (Becton Dickinson Immunocytometry Systems). Results are expressed as the percentage of positive cells.

### Tumor cell culture

The LNCaP cells were obtained from American Type Culture Collection (Rockville, MD) and cultivated in RPMI 1640 medium supplemented with 10% FBS, streptomycin 100 mg/mL, and penicillin 100 U/mL at 37°C in a 5% CO_2 _humidified incubator.

### Tumor RNA preparation

LNCaP cells cultivated in RPMI-1640 with 10% FBS were harvested and maintained at -80°C until RNA extraction. The RNA was extracted with Trizol (Invitrogen, Carlsbad, USA) following the supplier's recommendations. RNA concentration and purity were estimated in a 260/280 nm spectrophotometer. RNA integrity was verified using an Agilent 2100 Bioanalyzer (Agilent technologies, CA, USA).

### Dendritic cell transfection with LNCaP tumor cell RNA

Boczkowski *et al*. [11] suggested that RNA introduction alone activates DCs and causes them to mature. Based on this knowledge, we tested mature and immature DC transfection. Suspensions of DCs (4 × 10^6 ^cells/mL) were harvested at the 5^th ^(immature cells) and 7^th ^(mature DC) day of culture after TNF-α addition. Different conditions of transfection were used. For mature DCs, 4 μg and 5 μg RNA were electroporated with 300, 400, and 500 V pulses; additionally, we incubated cells with 5 μg RNA. For immature DCs, 4 μg and 16 μg were used for transfection with 300 and 400 V pulses. The electroporation was performed in a 4-mm curvette at 25 μF capacitance.

### Kinetics of tumor antigen expression

The expression of the tumoral antigen PSA was examined 24 h after transfection via immunocytochemistry with antibodies to PSA and the androgen receptor (Dako, Glostrup, Denmark). Briefly, DCs were recovered from culture plates using a cell scraper. Cells were fixed in 70% alcohol and submitted to 900 g centrifugation for 5 min at room temperature. The cytocentrifugate were impressed on adhesive coated slides and incubated overnight at 4°C with monoclonal antibodies to PSA or the androgen receptor at a dilution of 1:50 in PBS. Then, biotinylated anti-mouse immunoglobulin G was applied at a 1:200 dilution for 60 minutes at room temperature. Slides were rinsed with PBS for 30 minutes, incubated with peroxidase-conjugated streptavidin (streptABC Kit, Dako) at a 1:400 dilution in PBS for 45 minutes at room temperature, and rinsed with PBS for 30 minutes. Color was developed by incubating the slides in 0.06% diaminobenzidine in PBS for 15 minutes. Slides were then rinsed in tapwater, counterstained with Harris hematoxylin, dehydrated, coverslipped, and reviewed under a light microscope.

## Results

No single marker is exclusively present on the DC surface. Therefore, DC characterization requires the investigation of several cell surface markers [12]. In this study, these markers included CD80, CD86, CD14, CD11c, and CD1a; expression levels were measured on the 5th and 7th days of monocyte cell culture. All observed values are displayed at Table 1.

**Table 1:** Phenotypic characterization of immature and mature DCs by flow-cytometry.

**Membrane markers**	**Immature DC**	**Mature DC**
CD80	11.5%	48.4%
CD86	65.2%	82.4%
CD11c	84.9%	88.1%
CD14	0.59%	0.48%
CD1a	49.9%	97.9%
CCR7	1.75%	50.5%

Phenotype determination was performed by two color immunostaining using combinations of FITC- and PE-labeled mAbs. Cells (1 × 10^6^) were resuspended in PBS containing bovine serum albumin 0.1% and then incubated for 20 min at 4°C with optimal concentrations of the monoclonal antibodies. Membrane markers were determined by flow cytometry, and data from 10,000 events in mononuclear cell gates were collected and analyzed by Cell Quest Pro software. Results are expressed as the percentage of positive cells.

Monocyte cell cultures were obtained after apheresis and cultivated with cytokines (GM-CSF, IL-4, and TNF-α). We observed high levels of adhesion molecule CD11c (a myeloid blood DC marker) and low expression of monocyte/macrophage marker CD14 at both the 5^th ^and 7^th ^days, before and after TNF-α addition, respectively (Table 1). These expression patterns confirm a low percentage of monocytes at both cell culture stages.

DC maturation was characterized by an augmentation in the expression of the costimulatory molecules CD80 and CD86 (Table 1). At the 5^th ^day of differentiation, cells presented low levels of CD80 (a maturation marker) and CD86 (an early maturation marker). Furthermore, the percentage of cells expressing CD1a and CCR7 was also low. These expression levels are in accordance with the pattern observed for immature DCs. At this point, TNF-α was added to the culture medium and caused DC maturation as indicated by the increased expression of CD80, CD86, CD1a, and CCR7 at the 7^th ^day. Immature (5^th ^day) and mature (7^th ^day) DCs were used for RNA transfections.

**Figure 1:**
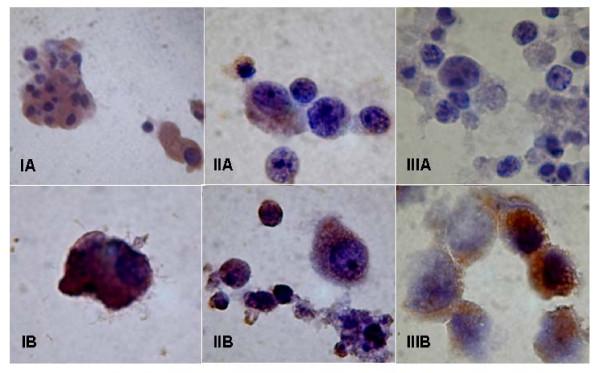
**PSA antigen expression of immature DCs transfected with total RNA from LNCaP cells**. Immature DCs were transfected by electroporation with 4 μg (IA and IB) and 16 μg (IIA and IIB) of RNA from LNCaP cells. The electroporation conditions were 300 (IA and IIA) or 400 V (IB and IIB). In both cases, the capacitance was 25 μF. IIIA is the negative transfection control, and IIIB is the positive control represented by LNCaP. The panel of photographs represents PSA expression detected by immunocytochemistry using an anti-PSA antibody.

**Figure 2:**
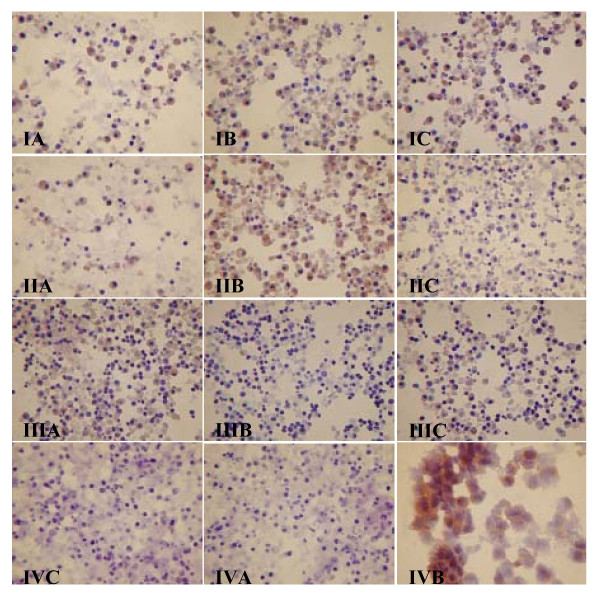
**PSA antigen expression of mature DCs transfected with total RNA from LNCaP cells**. Mature DCs were transfected by electroporation with 4 μg (IA, B, and C) and 5 μg (IIA, B, and C) or coincubated with 5 μg (IIIA, B, and C) of RNA from LNCaP cells. The electroporation conditions were 300 (IA and IIA), 400 (IB and IIB), or 500 V (IC and IIC). In all cases, the capacitance was 25 μF. The transfection by coincubation was done without preincubation (IIIA) and with 30 minutes (IIIB) or 2 hours (IIIC) of preincubation at 22°C. Photos IVA and IVB are negative transfection controls, and IVC is the positive control represented by LNCaP cells. The panel of photographs represents PSA expression detected by immunocytochemistry using an anti-PSA antibody.

**Figure 3:**
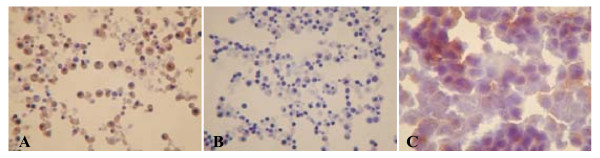
**Androgen receptor expression after mature DC transfection with total RNA from LNCaP cells**. (A) Mature DCs were transfected with 5 μg of LNCaP total RNA using 400 V electroporation and a capacitance of 25 μF. (B) Negative control of transfection represented by DCs, and (C) Positive control represented by LNCaP cells.

The efficiency of RNA transfection into DCs was investigated by immunocytochemistry using an anti-PSA antibody. All but one condition used for DC vaccine preparations resulted in PSA expression, as shown in Figures 1 (immature DCs) and 2 (mature DCs). A negative PSA assay was observed for one co-incubation condition (Figure 2-IIIB). Overall, the best transfection efficiency was observed for mature DC transfected via 400 V pulse electroporation with 5 μg total RNA (Figure 2-IIB). Expression of the androgen receptor was also positive, confirming the success of this strategy (Figure 3).

## Discussion

Immune responses depend on a variety of cellular processes, including transport of the MHC complex to the antigen presenting cell surface and its recognition by T-cell receptors as non-self. The core of these activities, however, is the MHC-peptide ligation. This ligation occurs in a restricted manner, in which each peptide binds only to its appropriate MHC. Thus, the use of selected peptides to pulse DC might fail because of the absence of specific MHCs. In Caucasians, HLA-A2 is frequent and motivates scientists to focus on HLA-A2-restricted peptides [13,14]. For other populations, this approach might not be an option because of the high genetic diversity observed. This is the case for the Brazilian population, which consists of three main ethnic groups (Caucasian, Black, and Amerindians). Furthermore, the Brazilian racial pattern is characterized by extensive miscegenation [15,16]. For this reason, we believe that mRNA molecules are the best choice for preparing therapeutic DC vaccines.

Different strategies have been proposed to transfect RNA into monocyte derived DCs, including electroporation and lipofection and others are still in development. Improvement of DC loading with RNA is an important issue for DC vaccine. RNA has the advantages of an efficient cytoplasmic expression allowing the use of total tumor antigens repertoire, and safe, because of its transient expression allied to non integrative properties into the host genome (for a review see [17]).

In our experiments, RNA molecules were introduced into immature and mature DCs, allowing both to be used in trials. Previous studies in prostate patients have shown that immune responses were initiated by vaccines with immature [18] and mature [19] DCs. However, a comparison study shows that the latter were superior to the former in the induction of immunological responses in melanoma patients [20]. The same conclusion was presented by McIlroy and Gregoire [21], who showed correlation between TNF and favorable responses in meta-analyses of ten clinical trials in melanoma patients (167 patients total). These data support mature DCs as the best choice for conducting clinical trials.

We prepared DC vaccines using allogeneic DCs transfected with cell culture RNA. Since MHC is highly polymorphic, there is a considerable possibility of MHC-mismatch among patients and leukapheresis donors. This difference can provoke immune responses against donor DCs instead of the tumor proteins that they carry. This problem could be solved by using patient peripheric blood to generate DCs. Most cancer patients, however, are already immunocompromised and debilitated due to previous treatment. Moreover, one tumor escape mechanism involves the inactivation of DC function. Thus, the use of patients' blood to obtain DCs may not be an option in all cases. Clinical observations, on the other hand, have shown that autologous DCs might be unnecessary; three patients negative for HLA-A2 were able to initiate immune responses after vaccination with HLA-A2 DCs loaded with PSMA peptide [22]. We have also observed a recovery of patients' immunological functions after two to three doses of DC vaccination [23]. Therefore, a viable possibility involves using allogeneic DCs for only the initial doses and then changing to more efficient vaccines prepared with autologous DCs.

## Conclusion

We believe that the treatment of patients with prostate metastatic disease using immunotherapy based on DCs is feasible in highly genetically polymorphic populations. For a more efficient immune response, entire mRNA molecules should be used instead of small peptides because of the diversity of the HLA molecules found in these populations. Furthermore, vaccines for at least the first two doses should be prepared with allogeneic DCs since patient immune systems may be suppressed by the tumor. Then, periferic blood of patients could be collected to prepare vaccines containing patients' own HLA molecules.

## Abbreviations

DC, Dendritic Cell, PSA, prostate-specific antigen.

## Competing interests

The author(s) declare that they have no competing interests.

## Authors' contributions

JMSC carried out the dendritic cell culture and transfection experiments and drafted the manuscript. FCC participated in the design of the study and helped to draft the manuscript. KRML analyzed the immunocytochemistry results and together with LHCL, coordinated the study. All authors read and approved the final manuscript.
